# Gender and Educational Trends in Lifetime Risk, Age at Onset, Expectancy, and Survival With Cardiovascular Disease in Finland, 1996–2020

**DOI:** 10.1093/geronb/gbaf007

**Published:** 2025-01-25

**Authors:** Shubhankar Sharma, Pekka Martikainen, Mikko Myrskylä, Lasse Tarkiainen, Ulla Suulamo

**Affiliations:** Helsinki Institute for Demography and Population Health, Faculty of Social Sciences, University of Helsinki, Helsinki, Finland; Max Planck–University of Helsinki Centre for Social Inequalities in Population Health, Helsinki, Finland; Max Planck–University of Helsinki Centre for Social Inequalities in Population Health, Rostock, Germany; Helsinki Institute for Demography and Population Health, Faculty of Social Sciences, University of Helsinki, Helsinki, Finland; Max Planck–University of Helsinki Centre for Social Inequalities in Population Health, Helsinki, Finland; Max Planck–University of Helsinki Centre for Social Inequalities in Population Health, Rostock, Germany; Helsinki Institute for Demography and Population Health, Faculty of Social Sciences, University of Helsinki, Helsinki, Finland; Max Planck–University of Helsinki Centre for Social Inequalities in Population Health, Helsinki, Finland; Max Planck–University of Helsinki Centre for Social Inequalities in Population Health, Rostock, Germany; Max Planck Institute for Demographic Research, Rostock, Germany; Helsinki Institute for Demography and Population Health, Faculty of Social Sciences, University of Helsinki, Helsinki, Finland; Max Planck–University of Helsinki Centre for Social Inequalities in Population Health, Helsinki, Finland; Max Planck–University of Helsinki Centre for Social Inequalities in Population Health, Rostock, Germany; Helsinki Institute for Demography and Population Health, Faculty of Social Sciences, University of Helsinki, Helsinki, Finland; International Max Planck Research School for Population, Health and Data Science, Rostock, Germany; (Social Sciences Section)

**Keywords:** Health disparities, Morbidity, Mortality, Multistate modeling

## Abstract

**Objectives:**

Cardiovascular disease (CVD) is the leading cause of mortality globally. Examining trends in CVD burden and associated sociodemographic disparities can contribute to tailoring policies that promote cardiovascular health and narrow health disparities. However, existing studies predominantly focus only on mortality. Therefore, we provide a more comprehensive understanding of CVD trends by studying the diverse aspects of CVD burden: lifetime risk, onset age, CVD-free and CVD life expectancy, and survival with CVD. We focus on the overall Finnish population in 1996–2020, as well as gender and educational disparities.

**Methods:**

We use sociodemographic information from individual-level population registers, which are linked to hospital discharge and Death Registers, on the entire Finnish population aged 40–100 years in five five-year periods in 1996–2020 (*N* = 2,796,732–3,273,232). We employed multistate models to derive the study metrics.

**Results:**

Overall, CVD’s lifetime risk stabilizes at 72% following a rise, with onset age and CVD-free life expectancy increased by 3 years. Although men bear a higher CVD burden, they experience a greater increase in onset age and CVD-free expectancy than women. Educational disparities in CVD-free expectancy persist, exceeding 3.5 years for men and women. Furthermore, survival with CVD has extended by 2.8 years but educational disparities widen.

**Discussion:**

Despite the encouraging CVD trends in the overall population and progress in narrowing gender disparities, there remains considerable room for further improvement. Persistent educational disparities in CVD burden underscore the need for more effective interventions to address enduring inequalities.

The cardiovascular disease (CVD) epidemic started in the United States in the 1930s and reached European countries after World War II (Levi et al., 2002; Williams, 1995). Particularly, in Finland, CVD mortality began rising in the 1950s, reaching the world’s highest level in the late 1960s (Williams, 1995). The implementation of community-based intervention programs in the 1970s, along with advancements in secondary and tertiary prevention during the 1980s and 1990s, decreased CVD mortality remarkably in Finland (Graham et al., 2007; Jousilahti et al., 2016; Laatikainen et al., 2005; Mähönen et al., 2014). Despite such progress, CVD remains the leading cause of mortality in high-income countries, posing serious public health problems (Eurostat, 2023; Levi et al., 2002).

There is an urgent need to keep track of the levels and trends in the burden of CVD, especially in high-income countries like Finland for several reasons. First, declined CVD mortality over recent decades means more adults are now living with CVD than ever before in Finland, increasing the number of adults suffering from its adverse consequences (Jousilahti et al., 2016; Levi et al., 2002). CVD is associated with multidimensional ramifications, affecting cognitive function, physical health, and quality of life of the adults living with CVD (Klijs et al., 2011; Liang et al., 2021; Saarni et al., 2006). Adults affected by CVD are also at increased risk of unemployment and spend less time in the labor market (Kruse et al., 2009; Maaijwee et al., 2014).

Second, Finland has the fastest-aging population in Europe and is a global leader in aging (Pirhonen et al., 2020). The share of older adults (aged 65+) in the population has surged from 14.8% in 2000 to 23.4% in 2023 (Official Statistics of Finland, 2024). By 2060, one in three individuals in Finland will be an older adult (National Institute for Health and Welfare, 2018). Since age is a strong risk factor for CVD (Hyvärinen et al., 2010), the rapid population aging in Finland is substantially growing the share of the population at an increased risk of CVD.

Third, despite progress in reducing CVD risk factor levels over the past decades (Borodulin et al., 2015), the percentage of adults with an ideal cardiovascular health profile, defined by having positive behavioral, biological, and physiological risk factors, remains considerably low in Finland (Peltonen et al., 2014). This is especially concerning given that the ideal cardiovascular health profile prevalence decreases with age, with the prevalence also being substantially lower among older men (Peltonen et al., 2014). Population aging, especially the greater expansion of the share of older men due to their faster increase in total life expectancy (TLE), is likely to decrease the ideal cardiovascular health profile prevalence further. This could lead to an increase in CVD cases, placing strain on the health and social care system, and society more broadly.

Fourth, the ramifications of CVD extend to family members, who often serve as informal caregivers (Aggarwal et al., 2009). In Finland, most informal caregivers are women—partners or daughters, and a substantial proportion of informal caregivers are also of working age (Mikkola et al., 2021). Evidence shows that caregivers of CVD patients are themselves at increased risk of CVD morbidity and mortality (Aggarwal et al., 2009). Further, caregivers belonging to the workforce may face high levels of stress due to the competing demands of work and caregiving (Martire & Stephens, 2003).

Moreover, the number of adults living alone has doubled since 1990. Nearly half of the 1.3 million (out of a total population of 5.5 million) individuals living alone are aged 60 or older (Official Statistics of Finland, 2022). These trends raise concern about the availability of social support for a growing older population. Given the multidimensional impacts of CVD and the ongoing sociodemographic trends, it has become essential to closely monitor the evolution of the CVD burden, gaining deeper insights into the trends in multiple aspects of the burden.

The diverse range of aspects, including lifetime risk, onset age, life expectancy with and without morbidity, and survival with morbidity, provides a holistic understanding of the morbidity burden, reflecting its key facets (Cai & Lubitz, 2007; Mehta & Myrskylä, 2017). However, existing studies on CVD trends often focus solely on assessing mortality trends (Cheema et al., 2022; Jousilahti et al., 2016; Laatikainen et al., 2005; Vartiainen et al., 2010), offering a limited perspective on the change in the burden as the CVD burden does not only arise from mortality. Furthermore, examining trends in different aspects of the CVD burden would also help us discern which of the three key population health change scenarios, namely, morbidity compression, morbidity expansion, and dynamic equilibrium (Fries, 1980; Gruenberg, 1977; Manton, 1982), is unfolding for CVD. Such insights are crucial for understanding how the burden evolves in the context of increasing longevity.

The optimistic *morbidity compression* hypothesis suggests that the postponement of morbidity onset would exceed the TLE increase, leading to a shorter morbid period before death (Fries, 1980). In contrast, the pessimistic *morbidity expansion* hypothesis posits that technological advancement would increase TLE without postponing morbidity onset, increasing only morbid years (Gruenberg, 1977). The *dynamic equilibrium* theory proposes that with mortality reduction, morbidity onset will also be postponed, leading to an increase in both morbid-free and morbid years (Manton, 1982).

Therefore, using incidence-based multistate models on high-quality Finnish register data, this study provides novel insights into the trends in the CVD burden and contributes to the global population health literature in multiple key ways. First, we analyze the trends in the lifetime risk of CVD at age 40 in five five-year periods from 1996 to 2020. Second, we investigate the trends in the mean age at the onset of CVD. Third, we examine the trends in CVD and CVD-free expectancies at age 40. Fourth, we assess the trends in survival of adults with CVD in the older adult population (aged 65+), the subgroup with substantial CVD mortality risk (Wang et al., 2014). Specifically, we analyze the trends in TLE at age 65 for adults with CVD.

Furthermore, CVD is not uniformly distributed across the population, with men and the lower educated experiencing a substantially higher likelihood of CVD than their counterparts (Hyvärinen et al., 2010; Silventoinen et al., 2005). Studies also find variations in the trends in CVD mortality by gender and educational attainment in numerous European countries (Di Girolamo et al., 2020). Therefore, beyond the overall pattern, we also examine how the trends in the CVD burden vary across these key subpopulations. The findings from this paper hold high policy relevance as they may form the foundation for tailoring preventive strategies for subgroups with poorer cardiovascular health progress despite developments in CVD prevention and care over the past decades, thereby contributing to the reduction of health disparities.

## Method

### Data and Study Population

This study is based on individual-level data for the entire Finnish population from 1996 to 2020. We include adults aged 40–100 years on 31 December each year, as available in the population registers. We obtained CVD diagnosis and mortality information from registers of inpatient hospital care and the Death Register, respectively. Using personal identification numbers of all the permanent residents in Finland, Statistics Finland links information from population registers to morbidity and mortality registers. We choose age 40 as the starting age as educational attainment is well established by this age. We divide 1996–2020 into five five-year periods: 1996–2000, 2001–2005, 2006–2010, 2011–2015, and 2016–2020.

To calculate the metrics of lifetime risk, onset age, and CVD and CVD-free expectancies, we use a well-established incidence-based discrete-time Markov chain multistate model, which has been applied in health research (Cai & Lubitz, 2007; Mehta & Myrskylä, 2017). This approach is used with data where individuals can transition between different health states over discrete time steps, although some states, such as the state of death, are irreversible once reached. To calculate these metrics in a multistate framework, we measure age in months, which ranges from 480 to 1,200 months, with a 3-month interval between consecutive ages. This approach is chosen because using yearly data might miss many first CVD events (i.e., hospitalization) that coincide with the year of death in Markov state assignments. The final analytical dataset consists of 4,264,350 adults aged 480–1,200 months (i.e., 40–100 years) contributing 275,523,409 quarterly-spaced observations from 1996 to 2020. The analysis on the survival of those with CVD focuses on older adults (i.e., aged 65–100), with 2,278,795 adults aged 780–1200 months contributing 94,777,481 quarterly-spaced observations from 1996 to 2020. We present the results in years for easier interpretation.

This study has been approved by the Institutional Review Board of the authority providing the pseudonymized data.

### Outcome variable

The outcome variable consists of three states, combining CVD and vital status: *CVD-free*, *CVD,* and *death*. These three states form the Markov state space (see Supplementary Figure 1).

### Cardiovascular disease

We classify adults with ischemic heart disease (IHD), heart failure, cerebrovascular disease, atrial fibrillation, or/and peripheral arterial disease as having CVD if they had any of these causes as their main diagnosis for the hospital episode (Korhonen et al., 2023). In Supplementary Table 1, we provide these diseases’ exact International Classification of Diseases codes. Although the study period is 1996–2020, we utilize information from 1970 (hospital discharge registers have data since 1970) to ascertain that those CVD-free in 1996–2020 did not also have a diagnosis in 1970–1995.

### Sociodemographic characteristics

Information on the sociodemographic variables of age, gender, and educational attainment is obtained from the Finnish total population registers. *Age* is measured in months. *Gender* is a binary variable (women and men). *Educational attainment* is the highest level of education obtained: basic education (International Standard Classification of Education, ISCED 0-2), secondary education (ISCED 3-4), and tertiary education (ISCED 5-8) (Korhonen et al., 2023). The categorical variable *period* represents the five five-year periods from 1996 to 2020.

### Analysis

We use Markov chain multistate models to derive the study metrics: lifetime risk, mean age at onset, and expectancies (Kemeny & Snell, 1983; Mehta & Myrskylä, 2017). This approach enjoys a wide range of advantages over the commonly applied prevalence-based Sullivan method. As the Sullivan method is based on morbidity prevalence, it represents a portion of the past risk of morbidity in the population and also does not allow mortality to vary by health status. In contrast, as the multistate method uses incidence data, it measures the current risk of CVD in the population. It also allows mortality to vary by CVD status. The lifetime risk of CVD is defined as the probability of ever experiencing CVD (Kemeny & Snell, 1983). The central inputs to the calculation are the transition probabilities.

To calculate the transition probabilities, we use multinomial logistic regressions (Allison, 1982), commonly applied in multistate research (Cai & Lubitz, 2007; Mehta & Myrskylä, 2017). For the total population, we use the following model:


$$\begin{array}{l} \log\frac{p_{ij}}{p_{iH}}=a_{ij}+\;b_{1,ij}Age+\;b_{2,ij}Age^{2}+\;{\delta_{ij}}^{T}\;Period \end{array}$$

where *p*_*ij*_ is the transition probability from state *i* to *j*. The state *j* also includes the state *death*; *H* is the reference state of *CVD*; $a_{ij}$ is the intercept; and $\delta_{ij}\;$ is a coefficient vector for the categorical period variable.

We next use the above-specified multinomial logistic regression model to calculate the age-specific transition probabilities for each of the eight subpopulations separately. These subpopulations are (a) all women, (b) all men, women with (c) basic, (d) secondary, and (e) tertiary education, and men with (f) basic, (g) secondary, and (h) tertiary education. To calculate the transition probabilities by gender, we do not control for educational attainment, and vice-versa. This is because we focus primarily on understanding the trends for men and women with different educational levels (and vice versa), as they actually exist in a population.

To obtain the probabilities for the first period, we set the first period (i.e., 1996–2000) to 1 and the rest of the periods to 0. Similarly, we obtain the transition probabilities for the remaining periods. In Supplementary Figures 2 and 3, we also show the age-specific probabilities for two key transitions: CVD-free to CVD and CVD to death. The transition probabilities are then inserted into the Markov transition matrix, from which we obtain the study metrics using the standard approach (Schneider, 2023). A detailed multistate calculation procedure is provided in Supplementary Material. To understand the trends in survival of older adults with CVD, we examine the trends in TLE at age 65 for those with CVD. We perform these calculations using the *dtms* package in STATA 17 (Schneider, 2023).

## Results

Table 1 presents the characteristics of the study population across the periods. The population’s mean age has increased by 3–4 years, reflecting population aging. The population’s educational profile has improved: the share of the highest educated women (men) has increased by about 17 (9) percentage points. Men, who have greater CVD prevalence, experience a greater increase in prevalence than women (7 vs 5 percentage points).

**Table 1.** Characteristics of the Study Population at Each Period

Variable	1996–2000	2001–2005	2006–2010	2011–2015	2016–2020
*Men*					
Mean age (years)	56.6	57.4	58.4	59.6	60.6
Educational attainment (%, *N*)					
Tertiary	23.0 (300,315)	25.4 (351,996)	27.6 (402,378)	29.8 (449,096)	31.7 (496,390)
Secondary	29.7 (387,444)	33.7 (467,789)	36.6 (533,517)	38.7 (584,199)	40.6 (637,700)
Basic	47.3 (617,939)	40.9 (567,587)	35.8 (521,708)	31.5 (475,757)	27.7 (434,864)
CVD prevalence (%)	18.9	21.1	23.0	24.7	25.9
*Women*					
Mean age (years)	59.8	60.3	61.0	62.0	62.8
Educational attainment (%, *N*)					
Tertiary	21.1 (315,024)	25.5 (397,244)	29.7 (480,476)	33.7 (559,076)	37.6 (641,354)
Secondary	27.8 (414,079)	30.8 (480,702)	32.9 (531,671)	34.2 (567,515)	35.4 (602,808)
Basic	51.1 (761,931)	43.7 (681,540)	37.4 (605,403)	32.1 (532,881)	27.0 (460,116)
CVD prevalence (%)	16.0	17.5	18.8	20.0	20.8
Total observations	49,914,715	52,916,030	55,783,886	57,609,462	59,299,316
*N*	2,796,732	2,946,858	3,075,153	3,168,524	3,273,232

*Note*: CVD = cardiovascular disease.

### Trend in the lifetime risk of cardiovascular disease

Table 2 presents the trends in CVD’s lifetime risk at age 40. Overall, the lifetime risk increased by 3 (72%–69%) percentage points between 1996–2000 and 2006–2010. However, it remains stable at 72% thereafter. Men’s lifetime risk is higher than women’s (e.g., 73% vs 71% in 2016–2020). This gender disparity has increased from 1 to 2 percentage points across the periods due to a greater increase in men’s lifetime risk.

**Table 2.** Trends in the Lifetime Risk (in %) of Cardiovascular Disease at Age 40 for Overall Population and by Gender and Educational Attainment in Finland

Periods	Total	Women	Men	Women			Men		
				Basic	Secondary	Tertiary	Basic	Secondary	Tertiary
1996–2000	69	68	69	71	63	58	71	66	68
2001–2005	71	70	71	73	66	61	72	68	70
2006–2010	72	71	72	74	67	62	74	68	71
2011–2015	72	71	73	74	67	63	74	69	71
2016–2020	72	71	73	74	67	63	74	69	72

The lifetime risk is the highest for adults with basic education, irrespective of gender and period (e.g., 74% in 2016–2020). The basic educated women experience a smaller increase in lifetime risk than the highest educated across the periods, narrowing the disparity by 2 percentage points. However, this disparity among men has been persistent at 2–3 percentage points.

### Trend in the mean age at onset of cardiovascular disease

**Figure 1.**
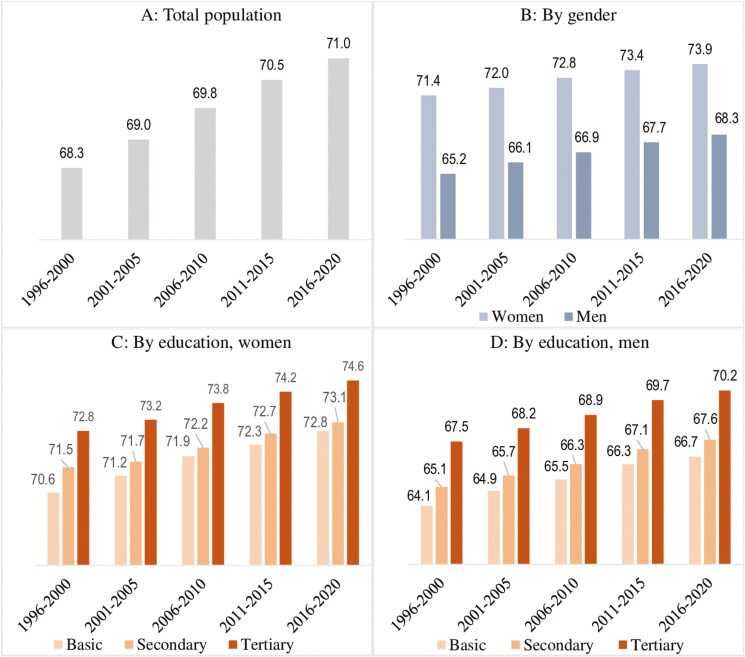
Trends in the mean age at onset of cardiovascular disease for the overall population (**A**), by gender (**B**), and by educational attainment among women (**C**) and men (**D**) in Finland.

Panels A–D in Figure 1 present the trends in the mean age at CVD’s onset. The age at onset for the overall population has increased from 68.3 to 71.0 years. Men persistently experience an earlier onset of CVD than women (e.g., 5.6 years earlier in 2016–2020). This gender disparity has declined from 6.2 to 5.6 years due to a greater onset postponement for men.

Adults with basic education experience the earliest onset, with secondary education lying between the basic and tertiary education. For instance, in 2016–2020, basic and secondary-educated men (women) experienced the onset 3.5 (1.8) and 2.6 (1.5) years earlier than their tertiary-educated counterparts, respectively. Basic educated women, however, experience a greater onset postponement than the highest educated, narrowing the disparity from 2.2 to 1.8 years. In contrast, this educational disparity has widened slightly from 3.4 to 3.5 years among men.

### Trends in expectancies at age 40

Table 3 presents the trends in CVD and CVD-free expectancies at age 40 for the overall population, by gender, and educational attainment. Overall, the TLE in 1996–2000 was 38.3 years: 30.5 years were CVD-free and 7.8 years with CVD. In 2016–2020, TLE increased to 42.5 years: 33.3 years were CVD-free and 9.2 years with CVD. Thus, about 67% or 2.8 years of the 4.2 life years added are spent CVD-free. Overall, the percentage of TLE spent CVD-free decreased by 2 percentage points between 1996–2000 and 2006–2010 and remained stable at 78% thereafter.

**Table 3.** Trends in the Expectancies (in years) At the Age of 40 in Finland

Variable	Period					Women				Men			
		TLE	CVD-free	CVD	% CVD-free	TLE	CVD-free	CVD	% CVD-free	TLE	CVD -free	CVD	% CVD-free
Total population	1996–2000	38.3	30.5	7.8	80	41.3	33.9	7.4	82	35.1	27.0	8.1	77
	2001–2005	39.6	31.1	8.5	79	42.4	34.4	8.0	81	36.7	27.9	8.8	76
	2006–2010	40.9	32.0	8.9	78	43.6	35.2	8.4	81	37.9	28.7	9.2	76
	2011–2015	41.7	32.7	9.0	78	44.3	35.8	8.5	81	39.2	29.7	9.5	76
	2016–2020	42.5	33.3	9.2	78	44.9	36.3	8.6	81	40.0	30.3	9.7	76
Education attainment													
Basic	1996–2000					39.9	32.6	7.3	82	33.3	25.4	7.9	76
	2001–2005					40.9	32.9	8.0	80	34.7	26.1	8.6	75
	2006–2010					42.2	33.7	8.5	80	35.8	26.8	9.0	75
	2011–2015					42.7	34.1	8.6	80	36.8	27.6	9.2	75
	2016–2020					43.2	34.5	8.7	80	37.5	28.1	9.4	75
Secondary	1996–2000					42.2	34.6	7.6	82	35.8	27.2	8.6	76
	2001–2005					43.2	34.7	8.5	80	37.1	27.8	9.3	75
	2006–2010					44.1	35.1	9.0	80	38.0	28.5	9.5	75
	2011–2015					44.6	35.6	9.0	80	39.2	29.4	9.8	75
	2016–2020					45.0	36.0	9.0	80	39.9	29.9	10.0	75
Tertiary	1996–2000					43.6	36.7	6.9	84	39.0	30.1	8.9	77
	2001–2005					44.6	36.9	7.7	83	40.3	30.8	9.5	76
	2006–2010					45.6	37.3	8.3	82	41.4	31.5	9.9	76
	2011–2015					46.4	37.8	8.6	81	42.5	32.3	10.2	76
	2016–2020					47.0	38.2	8.8	81	43.3	32.9	10.4	76

*Notes*: CVD = cardiovascular disease; TLE = total life expectancy.

Men experience a greater increase in TLE than women, narrowing the gender disparity from 6.2 to 4.9 years across the periods. Women have a considerably higher number and share of CVD-free years than men. However, the CVD-free expectancy increase has been greater for men than women, narrowing the gender gap from 6.9 to 6.0 years. CVD expectancies have been higher for men than women (e.g., 9.7 vs 8.6 years in 2016–2020). The greater increase in CVD expectancy for men has widened the gender disparity from 0.7 to 1.1 years.

TLE and CVD-free expectancy at age 40 for each period are the highest for tertiary-educated women and men and the lowest for the basic educated, with the secondary educated lying in the middle. The educational disparities in TLE have been persistent. For women, the disparity between the tertiary and basic education in CVD-free expectancy has narrowed from 4.1 to 3.7 years across the periods, whereas this disparity has been persistent at about 5 years for men. Despite having the shortest lives, basic educated women and men live a greater share of TLE with CVD than their tertiary-educated counterparts.

### Trends in survival of older adults with cardiovascular disease

**Figure 2.**
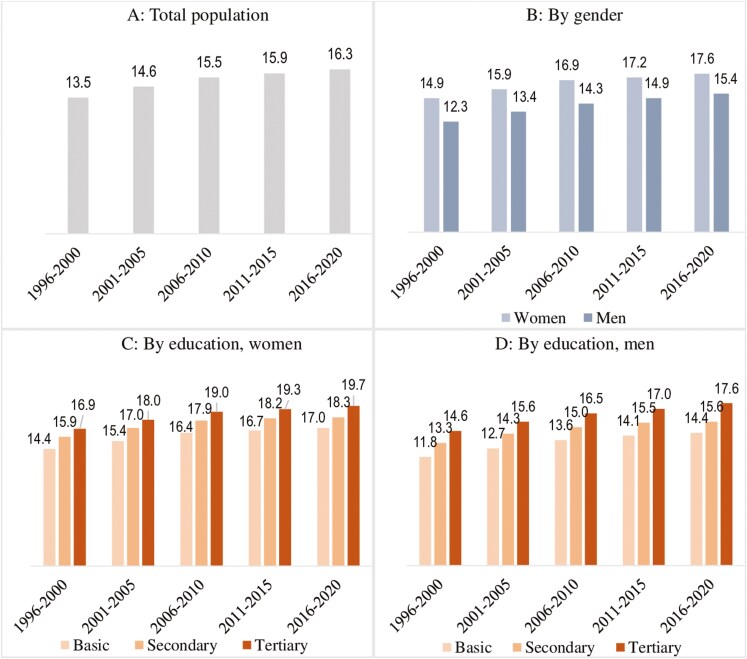
Trends in total life expectancy (in years) at age 65 for adults with cardiovascular disease for the overall population (**A**), by gender (**B**), and by educational attainment among women (**C**) and men (**D**) in Finland

Panels A–D in Figure 2 present the trends in survival with CVD in the older adult population. Mortality of adults with CVD refers to all-cause mortality (vs CVD mortality) as multistate models used allow for only one absorbing state. Overall, TLE at age 65 for adults with CVD has increased by 2.8 years across the periods. This increase is slightly greater for men than women, narrowing the gender disparity from 2.6 to 2.2 years. Stark educational disparities do exist, with basic educated men and women with CVD aged 65 having the shortest TLE. The increase in TLE for those with CVD is greater for tertiary-educated men (3.0 years) and women (2.8 years) compared with those with basic education whose life expectancy increased by 2.6 years, widening the disparities.

## Discussion

Examining the trends in CVD burden and their sociodemographic disparities has important public health implications, given the global challenge of CVD. It can provide valuable information to policymakers to guide the promotion of cardiovascular health by planning health interventions and may lay the foundations for developing policies that address the unique needs of vulnerable subpopulations. Therefore, using incidence-based discrete-time Markov chain multistate models based on quality Finnish register data covering the entire population, we contribute to the population health literature with a more nuanced and comprehensive understanding of the trends in CVD burden. We have five major findings:

First, the lifetime risk of CVD has remained stable at about 72% for the total population over recent decades. Second, adults have been able to postpone the onset by about three years in the past two decades. Third, about two-thirds of the TLE increase across the periods is CVD-free. These findings may reflect the favorable long-term trends observed in behavioral risk factors in Finland (i.e., lower cholesterol and blood pressure levels and reduced smoking prevalence) (Borodulin et al., 2015; Tolonen et al., 2023; Vartiainen et al., 2000), which have explained most of the CVD mortality reduction (Jousilahti et al., 2016; Vartiainen et al., 2010). To understand the role of the improved educational profile of the population, as observed in Table 1, on the trends in CVD burden, we performed additional analyses to study the education-adjusted trends in CVD burden in Supplementary Tables 2–5. Specifically, we calculate the age-specific transition probabilities for the study periods by setting the educational distribution to its proportion in 1996–2020 (i.e., accounting for the differences in educational attainment across study periods). We observe that the increases in mean age at onset of CVD, CVD-free life expectancy, and survival with CVD among older adults across the periods, as observed in the main part of the analyses, are attenuated after adjusting for education. For example, the CVD-free life expectancy increases for men (women) in education unadjusted and adjusted analyses are 3.3 (2.4) and 2.7 (1.9) years, respectively. These findings suggest that the encouraging CVD trends in Finland are partly attributable to the improved educational profile of the population over recent decades.

The fourth unique contribution of this study is the examination of trends in CVD burden with reference to two key determinants: gender and education. Despite longer exposure to advanced ages, women consistently have a lower CVD burden than men. However, men experience a greater onset postponement and CVD-free expectancy increase than women, narrowing the gender disparities slightly to about six years. A somewhat greater increase in men’s TLE and lifetime risk may lead to their greater CVD expectancy increase, marginally widening the disparities. The educational disparities in TLE at age 40 have been greater among men than women throughout the study periods, consistent with prior evidence (Martikainen et al., 2013). We observe similar patterns for the mean age at onset and expectancies with and without CVD. Although the least educated women experience a greater increase in the CVD onset age and CVD-free expectancy and a smaller increase in the lifetime risk than the highest educated women, their disparities with the highest educated women narrow only marginally. Somewhat worryingly, the disparities between the highest and the least educated men show no sign of narrowing.

The current study provides a more nuanced understanding of the evolution of the CVD burden along with the disparities in Finland. Exploring the mechanisms behind these disparities presents a promising avenue for further research, likely involving multifaceted explanations.

For instance, the lower CVD burden in women may be partly attributed to circulating estrogens providing a protective influence, and distinct genes regulating body fat distribution and insulin resistance, making men more susceptible to CVD (Charchar et al., 2003; McCarthy, 2007; Xiang et al., 2021). Women also have lower behavioral risk factor levels than men (Borodulin et al., 2015; Paalanen et al., 2020). However, Finnish men have experienced a remarkably greater decline in smoking prevalence over the past decades than women (Borodulin et al., 2015; Jousilahti et al., 2016). The increase in psychosocial risk factors of CVD like depression, anxiety, and stress in Finland has also been less prominent among men (Filatova et al., 2019; Sutela et al., 2019).

The inverse association between educational attainment and CVD is also well established, partly due to higher behavioral and medical risk factors among the less educated (Dégano et al., 2017; Silventoinen et al., 2005). Greater susceptibility to CVD among men and the low educated might combine to create a disproportionate CVD burden for the least educated men. Interestingly, compared with their highest-educated counterparts, basic-educated women have shown noticeable progress in cardiovascular health in terms of age at onset and CVD-free life expectancy. The favorable cardiovascular trends for the basic educated women may be partly attributed to beneficial risk factors trends. For instance, a study shows that the least educated women in Finland have experienced a significantly greater decline in risk factors like smoking, hypertension, obesity, and total cholesterol from 1997 to 2017 (Paalanen et al., 2020). In contrast, the decrease for the highest-educated women was found to be smaller and statistically insignificant (Paalanen et al., 2020). These differential trends in risk factor profile may also partly explain the differential trends in lifetime risk of CVD by educational attainment among women, with basic-educated women showing a smaller increase in lifetime risk compared with tertiary-educated women across the study periods.

The fifth distinctive aspect of this study involves exploring the trends in TLE of older adults with CVD and associated disparities. The considerable improvement in survival for older men and women with CVD can be partly attributed to the increasing impact of secondary prevention (reducing disease severity) and tertiary prevention (preventing complications through treatment) on CVD mortality reduction (Jousilahti et al., 2016; Laatikainen et al., 2005). The introduction of new secondary prevention guidelines in the 1980s, and advancements such as percutaneous coronary interventions in the 1990s, which increased fivefold between 1994 and 2011, have contributed to these positive trends (Graham et al., 2007; Mähönen et al., 2014). We find that the tertiary educated older adults with CVD experience a greater increase in TLE than the basic educated. The developments in secondary and tertiary preventions for CVD may benefit the socioeconomically advantaged more, contributing to increased inequality.

Monitoring health disparities is an integral part of the Finnish National Action Plan to narrow the disparities, which has been a major goal of Finnish health policy in the past 3 decades and elsewhere. Although there has been progress in reducing gender and educational disparities in CVD mortality in absolute terms (Di Girolamo et al., 2020), we find that the disparities in CVD morbidity have not narrowed considerably and, in certain aspects, they have even widened. Importantly, CVD’s lifetime risk has stabilized at a high level, with CVD expectancy nearing a decade, especially for men and the least educated. With substantial population aging and a high lifetime risk, the number of CVD cases is likely to increase in the coming years. The increasing number of cases with almost a decade-long CVD expectancy may have considerable ramifications at both individual and societal levels. CVD leads to a loss of over 64 million healthy life years and direct health care costs, informal care costs, and productivity losses for CVD add up to more than €200 billion per year in Europe (Wilkins et al., 2017). Consequently, the findings underscore the urgency of more effective preventive measures, especially tailored toward men and the least educated adults for an accelerated decline of their excessive CVD burden.

We can also analyze the evolving trends in CVD burden through the well-established framework of compression, expansion, and dynamic equilibrium of morbidity. Our findings of a postponed CVD onset and increases in both CVD and CVD-free expectancies, along with the increase in TLE, accord with *dynamic equilibrium* for CVD morbidity in Finland over recent decades. Achieving compression of CVD morbidity, which is desirable, will require a decline in CVD expectancy in the coming years. An encouraging development is that the pace of increase in CVD expectancy has slowed across the study periods. Nevertheless, continuous monitoring of these trends is warranted as the rates of key psychosocial risk factors of CVD, such as depression, anxiety, and stress have recently increased in Finland (Filatova et al., 2019; Sutela et al., 2019). Concerningly, the prevalence of obesity and diabetes, two major medical risk factors of CVD, is also projected to increase in Finland in the coming years (Tolonen et al., 2023).

### Strengths and limitations

This paper has several strengths. To the best of our knowledge, this study is the first in any nation to explore trends in CVD burden using a comprehensive array of key metrics, offering a multifaceted understanding of the burden across a 24-year period. Given that CVD is the leading cause of death globally, these insights are crucial for identifying disparities, allowing for the adaptation and targeting of preventive efforts to address contemporary needs.

The application of incidence-based multistate models to derive the study metrics, offering several advantages over the prevalence-based Sullivan method, is another distinctive strength. Unlike the Sullivan method, the multistate method leverages incidence data, reflecting the current CVD risk in the population, and allows differential mortality by health status and calculations of many key measures of CVD burden, such as lifetime risk and onset age. In short, the multistate approach helps to model contemporary cardiovascular health dynamics more accurately.

Whereas most studies on disease burden rely on sample data, which are usually exposed to selection bias or selective attrition, this study benefits from the use of unique and high-quality Finnish register data on the entire population, avoiding these biases. The prospective collection of CVD information based on hospital diagnoses also mitigates reporting or proxy reporting biases, adding another layer of strength to the paper. Finnish hospitalization and Death Registers are renowned for their reliability concerning CVD data (Sund, 2012) and have been intensively used in research (Korhonen et al., 2023; Silventoinen et al., 2005).

Nevertheless, this study has some limitations. The multistate calculation relies on the Markov assumption, which states that the transition probabilities depend only on the current state and covariates. The assumption, however, keeps the calculations tractable and is found to be a useful simplification in our context as we focus on calculating population-level quantities only, such as probabilities and means, not on predicting individual trajectories, for which the assumption may be problematic (Mehta & Myrskylä, 2017). Further, the transition probabilities also accurately represent the current CVD risk in the population.

Our results are based on a period perspective. We assume that the transition probabilities observed during a period (e.g., 2006–2010) remain constant throughout a synthetic cohort’s life. That is, the findings do not necessarily reflect the CVD burden for any actual birth cohort. Nevertheless, the period perspective helps us assess the social disparities in CVD burden in a straightforward manner and has been widely used in research (Cai & Lubitz, 2007; Mehta & Myrskylä, 2017). Further, we analyze CVD as a broad group of causes. This decision was motivated by the relatively long study period, which might be associated with diagnostic shifts between specific causes. Future studies should evaluate the morbidity trends for IHD and cerebrovascular disease, the most important CVD sub-categories.

## Conclusions

This study reveals encouraging trends in the cardiovascular health of the overall Finnish population over recent decades. Gender disparities in onset age and CVD-free expectancy are narrowing, indicating progress though with much room for improvement. However, persistent educational disparities in cardiovascular health progress remain worrying. Hence, strategic preventive measures are essential, extending beyond current implementations and focusing on men and least-educated adults, to narrow the disparities.

## Supplementary Material

gbaf007_suppl_Supplementary_Materials

## Data Availability

Statistics Finland and the Finnish Institute for Health and Welfare have collected and own the data. Due to data protection regulations, the authors are not allowed to make the data available to third parties. The authors have no special access privilege; all interested researchers can apply for data access by contacting the register-holding institutions: Statistics Finland (https://stat.fi/tup/tutkijapalvelut/index_en.html), by email tutkijapalvelut@stat.fi or by telephone + 358 29 551 2758, and the National Institute for Health and Welfare (https://thl.fi/en/research-and-development), mobile + 358 29 524 6000. For more information on the user licence process, please see https://stat.fi/tup/tutkijapalvelut/kayttoluvan-hakeminen-ja-lupamuutokset_en.html.

## References

[CIT0002] Aggarwal, B., Liao, M., Christian, A., & Mosca, L. (2009). Influence of caregiving on lifestyle and psychosocial risk factors among family members of patients hospitalized with cardiovascular disease. Journal of General Internal Medicine, 24(1), 93–98. https://doi.org/10.1007/s11606-008-0852-1 18998190 PMC2607516

[CIT0003] Allison, P. D. (1982). Discrete-time methods for the analysis of event histories. Sociological Methodology, 13, 61–98. https://doi.org/10.2307/270718

[CIT0004] Borodulin, K., Vartiainen, E., Peltonen, M., Jousilahti, P., Juolevi, A., Laatikainen, T., Männistö, S., Salomaa, V., Sundvall, J., & Puska, P. (2015). Forty-year trends in cardiovascular risk factors in Finland. European Journal of Public Health, 25(3), 539–546. https://doi.org/10.1093/eurpub/cku174 25422363

[CIT0005] Cai, L., & Lubitz, J. (2007). Was there compression of disability for older Americans from 1992 to 2003? Demography, 44(3), 479–495. https://doi.org/10.1353/dem.2007.0022 17913007

[CIT0006] Charchar, F. J., Tomaszewski, M., Strahorn, P., Champagne, B., & Dominiczak, A. F. (2003). Y is there a risk to being male? Trends in Endocrinology and Metabolism, 14(4), 163–8. https://doi.org/10.1016/s1043-2760(03)00032-8 12714276

[CIT0007] Cheema, K. M., Dicks, E., Pearson, J., & Samani, N. J. (2022). Long-term trends in the epidemiology of cardiovascular diseases in the UK: Insights from the British Heart Foundation statistical compendium. Cardiovascular Research, 118(10), 2267–2280. https://doi.org/10.1093/cvr/cvac053 35420124

[CIT0008] Dégano, I. R., Marrugat, J., Grau, M., Salvador-González, B., Ramos, R., Zamora, A., Martí, R., & Elosua, R. (2017). The association between education and cardiovascular disease incidence is mediated by hypertension, diabetes, and body mass index. Scientific Reports, 7(1), 12370. https://doi.org/10.1038/s41598-017-10775-3 28959022 PMC5620039

[CIT0009] Di Girolamo, C., Nusselder, W. J., Bopp, M., Brønnum-Hansen, H., Costa, G., Kovács, K., Leinsalu, M., Martikainen, P., Pacelli, B., Rubio Valverde, J., & Mackenbach, J. P. (2020). Progress in reducing inequalities in cardiovascular disease mortality in Europe. Heart, 106(1), 40–49. https://doi.org/10.1136/heartjnl-2019-315129 31439656 PMC6952836

[CIT0010] Eurostat. (2023). Causes of death—deaths by country of residence and occurrence. https://ec.europa.eu/eurostat/databrowser/view/hlth_cd_aro__custom_8852078/default/table?lang=en

[CIT0011] Filatova, S., Upadhyaya, S., Kronström, K., Suominen, A., Chudal, R., Luntamo, T., Sourander, A., & Gyllenberg, D. (2019). Time trends in the incidence of diagnosed depression among people aged 5-25 years living in Finland 1995-2012. Nordic Journal of Psychiatry, 73(8), 475–481. https://doi.org/10.1080/08039488.2019.1652342 31443615

[CIT0012] Fries, J. F. (1980). Aging, natural death, and the compression of morbidity. The New England Journal of Medicine, 303(3), 130–135. https://doi.org/10.1056/NEJM198007173030304 7383070

[CIT0013] Graham, I., Atar, D., Borch-Johnsen, K., Boysen, G., Burell, G., Cifkova, R., Dallongeville, J., De Backer, G., Ebrahim, S., Gjelsvik, B., Herrmann-Lingen, C., Hoes, A., Humphries, S., Knapton, M., Perk, J., Priori, S. G., Scholte op Reimer, W., Weissberg, P., Wood, D., … Sans-Menendez, S.; European Society of Cardiology (ESC) Committee for Practice Guidelines (CPG) (2007). European guidelines on cardiovascular disease prevention in clinical practice: Executive summary: Fourth Joint Task Force of the European Society of Cardiology and Other Societies on Cardiovascular Disease Prevention in Clinical Practice (Constituted by representatives of nine societies and by invited experts). European Heart Journal, 28(19), 2375–2414. https://doi.org/10.1093/eurheartj/ehm316 17726041

[CIT0014] Gruenberg, E. M. (1977). The failures of success. The Milbank Memorial Fund quarterly. Health and Society, 55(1), 3–24. https://doi.org/10.2307/3349592 141009

[CIT0015] Hyvärinen, M., Qiao, Q., Tuomilehto, J., Söderberg, S., Eliasson, M., & Stehouwer, C. D. (2010). The difference between acute coronary heart disease and ischaemic stroke risk with regard to gender and age in Finnish and Swedish populations. International Journal of Stroke, 5(3), 152–156. https://doi.org/10.1111/j.1747-4949.2010.00423.x 20536610

[CIT0016] Jousilahti, P., Laatikainen, T., Peltonen, M., Borodulin, K., Männistö, S., Jula, A., Salomaa, V., Harald, K., Puska, P., & Vartiainen, E. (2016). Primary prevention and risk factor reduction in coronary heart disease mortality among working aged men and women in eastern Finland over 40 years: Population based observational study. BMJ, 352, i721. https://doi.org/10.1136/bmj.i721 26932978 PMC4772739

[CIT0017] Kemeny, J. G., & Snell, J. L. (1983). Finite Markov chains. Springer-Verlag.

[CIT0018] Klijs, B., Nusselder, W. J., Looman, C. W., & Mackenbach, J. P. (2011). Contribution of chronic disease to the burden of disability. PLoS One, 6(9), e25325. https://doi.org/10.1371/journal.pone.0025325 21966497 PMC3178640

[CIT0019] Korhonen, K., Leinonen, T., Tarkiainen, L., Einiö, E., & Martikainen, P. (2023). Childhood socio-economic circumstances and dementia: Prospective register-based cohort study of adulthood socio-economic and cardiovascular health mediators. International Journal of Epidemiology, 52(2), 523–535. https://doi.org/10.1093/ije/dyac205 36343014 PMC10114069

[CIT0020] Kruse, M., Sørensen, J., Davidsen, M., & Gyrd-Hansen, D. (2009). Short and long-term labour market consequences of coronary heart disease: A register-based follow-up study. European Journal of Cardiovascular Prevention and Rehabilitation, 16(3), 387–391. https://doi.org/10.1097/HJR.0b013e32832a3333 19318953

[CIT0021] Laatikainen, T., Critchley, J., Vartiainen, E., Salomaa, V., Ketonen, M., & Capewell, S. (2005). Explaining the decline in coronary heart disease mortality in Finland between 1982 and 1997. American Journal of Epidemiology, 162(8), 764–773. https://doi.org/10.1093/aje/kwi274 16150890

[CIT0022] Levi, F., Lucchini, F., Negri, E., & La Vecchia, C. (2002). Trends in mortality from cardiovascular and cerebrovascular diseases in Europe and other areas of the world. Heart, 88(2), 119–124. https://doi.org/10.1136/heart.88.2.119 12117828 PMC1767229

[CIT0023] Liang, X., Huang, Y., & Han, X. (2021). Associations between coronary heart disease and risk of cognitive impairment: A meta-analysis. Brain and Behavior, 11(5), e02108. https://doi.org/10.1002/brb3.2108 33742562 PMC8119850

[CIT0024] Maaijwee, N. A., Rutten-Jacobs, L. C., Arntz, R. M., Schaapsmeerders, P., Schoonderwaldt, H. C., van Dijk, E. J., & de Leeuw, F. E. (2014). Long-term increased risk of unemployment after young stroke: A long-term follow-up study. Neurology, 83(13), 1132–1138. https://doi.org/10.1212/WNL.0000000000000817 25128177

[CIT0025] Mähönen, M., Pietilä, A., Havulinna, A. S., Koukkunen, H., Kärjä-Koskenkari, P., Juolevi, A., & Salomaa, V. (2014). Reliability and trends of register data concerning invasive procedures for coronary heart disease 1994–2011. Suomen Lääkärilehti, 69(33), 1953–1958.

[CIT0026] Manton, K. G. (1982). Changing concepts of morbidity and mortality in the elderly population. The Milbank Memorial Fund Quarterly. Health and Society, 60(2), 183–244. https://doi.org/10.2307/3349767 6919770

[CIT0027] Martikainen, P., Ho, J. Y., Preston, S., & Elo, I. T. (2013). The changing contribution of smoking to educational differences in life expectancy: Indirect estimates for Finnish men and women from 1971 to 2010. Journal of Epidemiology and Community Health, 67(3), 219–224. https://doi.org/10.1136/jech-2012-201266 23201620 PMC3886806

[CIT0028] Martire, L. M., & Stephens, M. A. P. (2003). Juggling parent care and employment responsibilities: The dilemmas of adult daughter caregivers in the workforce. Sex Roles, 48, 167–173. https://doi.org/10.1023/A:1022407523039

[CIT0029] McCarthy, J. J. (2007). Gene by sex interaction in the etiology of coronary heart disease and the preceding metabolic syndrome. Nutrition, Metabolism, and Cardiovascular Diseases, 17(2), 153–161. https://doi.org/10.1016/j.numecd.2006.01.005 17306735

[CIT0030] Mehta, N., & Myrskylä, M. (2017). The population health benefits of a healthy lifestyle: Life expectancy increased and onset of disability delayed. Health Affairs, 36(8), 1495–1502. https://doi.org/10.1377/hlthaff.2016.1569 PMC5775051 28724530

[CIT0031] Mikkola, T. M., Kautiainen, H., Mänty, M., von Bonsdorff, M. B., Kröger, T., & Eriksson, J. G. (2021). Age-dependency in mortality of family caregivers: A nationwide register-based study. Aging Clinical and Experimental Research, 33(7), 1971–1980. https://doi.org/10.1007/s40520-020-01728-4 33040307 PMC8249300

[CIT0032] National Institute for Health and Welfare. (2018). Statistical yearbook on social welfare and health care. http://www.julkari.fi/bitstream/handle/10024/137595/Sosiaali-%20ja%20terveysalan%20tilastollinen%20vuosikirja_2018_verkkoon.pdf?sequence=1&isAllowed=y

[CIT0034] Official Statistics of Finland (OSF). (2022). Dwellings and housing conditions [online publication]. Reference period: 2022. Statistics Finland [Referenced: 14.11.2024]. https://stat.fi/en/publication/cl8a30d0ruzs50cvv45kpapqg

[CIT0035] Official Statistics of Finland. (2024). Population structure [Online publication]. Statistics Finland. Retrieved November 13, 2024, from https://stat.fi/en/statistics/vaerak

[CIT0036] Paalanen, L., Härkänen, T., Kontto, J., & Tolonen, H. (2020). Inequalities by education and marital status in the co-occurrence of cardiovascular risk factors in Finland persisted between 1997–2017. Scientific Reports, 10(1), 9123. https://doi.org/10.1038/s41598-020-65959-1 32499541 PMC7272447

[CIT0037] Peltonen, M., Laatikainen, T., Borodulin, K., Wikström, K., Jousilahti, P., Jula, A., & Puska, P. (2014). Prevalence of ideal cardiovascular health in an adult Finnish population: The national FINRISK 2007 study. International Heart and Vascular Disease Journal, 2(3 (eng), 3–10.

[CIT0038] Pirhonen, J., Lolich, L., Tuominen, K., Jolanki, O., & Timonen, V. (2020). “These devices have not been made for older people’s needs”–Older adults’ perceptions of digital technologies in Finland and Ireland. Technology in Society, 62, 101287. https://doi.org/10.1016/j.techsoc.2020.101287

[CIT0039] Saarni, S. I., Härkänen, T., Sintonen, H., Suvisaari, J., Koskinen, S., Aromaa, A., & Lönnqvist, J. (2006). The impact of 29 chronic conditions on health-related quality of life: A general population survey in Finland using 15D and EQ-5D. Quality of Life Research 15(8), 1403–1414. https://doi.org/10.1007/s11136-006-0020-1 16960751

[CIT0040] Schneider, D. C. (2023). Discrete-time multistate regression models in Stata: The dtms module. https://www.stata.com/meeting/germany23/slides/Germany23_Schneider.pdf

[CIT0041] Silventoinen, K., Pankow, J., Jousilahti, P., Hu, G., & Tuomilehto, J. (2005). Educational inequalities in the metabolic syndrome and coronary heart disease among middle-aged men and women. International Journal of Epidemiology, 34(2), 327–334. https://doi.org/10.1093/ije/dyi007 15659460

[CIT0042] Sund, R. (2012). Quality of the Finnish Hospital Discharge Register: A systematic review. Scandinavian Journal of Public Health, 40(6), 505–515. https://doi.org/10.1177/1403494812456637 22899561

[CIT0043] Sutela, H., Pärnänen, A., & Keyriläinen, M. (2019). Digiajan työelämä− työolotutkimuksen tuloksia 1977−2018. *Statistics Finland*.

[CIT0044] Tolonen, H., Reinikainen, J., Zhou, Z., Härkänen, T., Männistö, S., Jousilahti, P., Paalanen, L., Lundqvist, A., & Laatikainen, T. (2023). Development of non-communicable disease risk factors in Finland: Projections up to 2040. Scandinavian Journal of Public Health, 51(8), 1231–1238. https://doi.org/10.1177/14034948221110025 35891611 PMC10642220

[CIT0045] Vartiainen, E., Jousilahti, P., Alfthan, G., Sundvall, J., Pietinen, P., & Puska, P. (2000). Cardiovascular risk factor changes in Finland, 1972-1997. International Journal of Epidemiology, 29(1), 49–56. https://doi.org/10.1093/ije/29.1.49 10750603

[CIT0046] Vartiainen, E., Laatikainen, T., Peltonen, M., Juolevi, A., Männistö, S., Sundvall, J., Jousilahti, P., Salomaa, V., Valsta, L., & Puska, P. (2010). Thirty-five-year trends in cardiovascular risk factors in Finland. International Journal of Epidemiology, 39(2), 504–518. https://doi.org/10.1093/ije/dyp330 19959603

[CIT0047] Wang, H., Sun, W., Ji, Y., Shi, J., Xuan, Q., Wang, X., Xiao, J., & Kong, X. (2014). Trends in age-specific cerebrovascular disease in the European Union. International Journal of Clinical and Experimental Medicine, 7(11), 4165–4173. https://pmc.ncbi.nlm.nih.gov/articles/PMC4276185/25550927 PMC4276185

[CIT0048] Wilkins, E., Wilson, L., Wickramasinghe, K., Bhatnagar, P., Leal, J., Luengo-Fernandez, R., Burns, R., Rayner, M., & Townsend, N. (2017). European cardiovascular disease statistics 2017. https://ehnheart.org/wp-content/uploads/2023/07/CVD-Statistics.pdf

[CIT0049] Williams, O. D. (1995). Total mortality and mortality from heart disease, cancer, and stroke from 1950 to 1987 in 27 countries: Highlights of trends and their interrelationships among causes of death. Annals of Epidemiology, 5(5), 417. https://doi.org/10.1016/1047-2797(95)90009-8

[CIT0050] Xiang, D., Liu, Y., Zhou, S., Zhou, E., & Wang, Y. (2021). Protective effects of estrogen on cardiovascular disease mediated by oxidative stress. Oxidative Medicine and Cellular Longevity, 2021, 5523516. https://doi.org/10.1155/2021/5523516 34257804 PMC8260319

