# Supplementary material for: Gender and Educational Trends in Lifetime Risk, Age at Onset, Expectancy, and Survival With Cardiovascular Disease in Finland, 1996–2020
Source: J Gerontol B Psychol Sci Soc Sci. 2025 Jan 25;80(4):gbaf007. doi: 10.1093/geronb/gbaf007 (PMC11949380; doi:10.1093/geronb/gbaf007)
Supplement: gbaf007_suppl_Supplementary_Materials [file gbaf007_suppl_supplementary_materials.docx]

***The Journals of Gerontology, Series B: Psychological Sciences and Social Sciences* Supplementary Material: Sharma et al. Gender and educational trends in lifetime risk, age at onset, expectancy, and survival with cardiovascular disease in Finland, 1996-2020.**

**Supplementary Table 1.** International Classification of Diseases 8th, 9th and 10th Revision codes for cardiovascular disease

| **Cardiovascular disease** | **ICD 8** | **ICD 9** | **ICD 10** |
| --- | --- | --- | --- |
| Ischaemic Heart Disease | 410–414 | 410–414 | I20–I25 |
| Cerebrovascular disease | 430–438 | 430–438 | I60–I69, G45 |
| Heart failure | 427.0, 427.1, 427.9 | 428 | I50 |
| Atrial fibrillation | 427.4 | 427.3 | I48 |
| Peripheral arterial disease | 440–447 | 440–447 | I70–I77 |

Note: ICD = International Classification of Diseases.

**Supplementary Table 2.** Education-adjusted trends in lifetime risk (in %) of cardiovascular disease at age 40 by gender in Finland

| Periods | Women | Men |  |
| --- | --- | --- | --- |
| 1996-2000 | 68 | 70 |  |
| 2001-2005 | 70 | 72 |  |
| 2006-2010 | 71 | 72 |  |
| 2011-2015 | 71 | 73 |  |
| 2016-2020 | 71 | 73 |  |

Supplementary Table 2 shows the education-adjusted trends in lifetime risk of CVD at age 40 for total women and men in Finland. The lifetime risk of CVD remains stable at 71% for women and 73% for men, after an increase in the initial periods.

**Supplementary Table 3.** Education-adjusted trends in mean age at onset of cardiovascular disease by gender in Finland

| Periods | Women | Men |
| --- | --- | --- |
| 1996-2000 | 71.8 | 65.7 |
| 2001-2005 | 72.3 | 66.4 |
| 2006-2010 | 73.0 | 67.0 |
| 2011-2015 | 73.4 | 67.8 |
| 2016-2020 | 73.8 | 68.3 |

Supplementary Table 3 presents the education-adjusted trends in mean age at onset of CVD. Across the periods, the mean ages at onset of CVD for women and men have increased by 2 and 2.6 years, respectively.

**Supplementary Table 4.** Education-adjusted trends in expectancies (in years) at age 40 by gender in Finland

| Period | Women | | | Men | | |
| --- | --- | --- | --- | --- | --- | --- |
|  | TLE | CVD-free | CVD | TLE | CVD-free | CVD |
| 1996-2000 | 41.9 | 34.5 | 7.4 | 36.0 | 27.6 | 8.4 |
| 2001-2005 | 43.0 | 34.9 | 8.1 | 37.3 | 28.2 | 9.1 |
| 2006-2010 | 44.1 | 35.5 | 8.6 | 38.4 | 28.9 | 9.5 |
| 2011-2015 | 44.6 | 36.0 | 8.6 | 39.5 | 29.8 | 9.7 |
| 2016-2020 | 45.1 | 36.4 | 8.7 | 40.2 | 30.3 | 9.9 |

*Note*: TLE = total life expectancy; CVD = cardiovascular disease.

Supplementary Table 4 presents the education-adjusted trends in expectancies at age 40. We find that TLE at age 40 for women and men have increased by 3.2 and 4.2 years across the periods, respectively. The corresponding increases in CVD-free life expectancy across the study periods are 1.9 and 2.7 years.

**Supplementary Table 5.** Education-adjusted trends in total life expectancy (in years) at age 65 for adults with cardiovascular disease by gender in Finland

| Period | Women | Men |
| --- | --- | --- |
|  | TLE | TLE |
| 1996-2000 | 15.0 | 12.6 |
| 2001-2005 | 16.0 | 13.6 |
| 2006-2010 | 17.0 | 14.5 |
| 2011-2015 | 17.3 | 15.0 |
| 2016-2020 | 17.6 | 15.3 |

*Note*: TLE = total life expectancy

Supplementary Table 5 presents the education-adjusted trends in survival with CVD in the older adult population. Across the periods, TLE at age 65 for women and men with CVD have increased by 2.6 years and 2.7 years, respectively.

**Calculations of the lifetime risk, average age at onset and expectancy with and without cardiovascular disease using discrete-time Markov chain multistate model:**

As described in the statistical analysis section, we use multinomial logistic regression models to obtain the transition probabilities across the states in the Markov state space by three-month age groups: 480, 483, 486, …, 1200. We insert these calculated transition probabilities into the Markov transition matrix below:

**M^T^** $=\left( \begin{matrix} \mathbf{p}_{\mathbf{NCVD}\mathbf{-}\mathbf{NCVD}} & \boldsymbol{0} & \mathbf{0} \\ \mathbf{p}_{\mathbf{NCVD}\mathbf{-}\mathbf{CVD}} & \mathbf{p}_{\mathbf{CVD}\mathbf{-}\mathbf{CVD}} & \mathbf{0} \\ \mathbf{p}_{\mathbf{NCVD}\mathbf{-}\mathbf{death}} & \mathbf{p}_{\mathbf{CVD}\mathbf{-}\mathbf{death}} & \mathbf{1} \end{matrix} \right)$

*NCVD* No cardiovascular disease

Each of the sub-matrices ($\mathbf{p}_{\mathbf{i-j}}$) in the Markov transition matrix (**M^T^**) is a 241 x 241 matrix (241: number of three-month age groups from 480 to 1200). Each sub-matrix contains non-zero numbers only on the first sub-diagonal. Each non-zero number represents the transition probability from state $\mathbf{i}$ to state $\mathbf{j}$ in an age transition. On the other hand, $\mathbf{p}_{\mathbf{NCVD-death}}$ and $\mathbf{p}_{\mathbf{CVD-death}}$ are row vectors of the order 1 x 241, that contain the probabilities of dying at an age. Each column of the matrix **M^T^** sums to one.

**Calculation of the lifetime risk and mean age at onset of cardiovascular disease:**

These calculations are based on the submatrices of **M^T^**. First, we calculate the probabilities of a 480-month-old adult free of cardiovascular disease (CVD) experiencing CVD for the first time after k time steps (k= number of three-month age groups in the range of 480 and 1200 month: 480, 483, 486, …, 1200): probability of experiencing CVD after first time step, probability of staying CVD-free for the first time step and experiencing CVD after second time step, probability of staying CVD-free for the first two time steps and experiencing CVD after third time step and similarly for all the 241 time steps. The calculation is as follows:

The probabilities of the transient state except for the CVD state in **M^T^** is:

**M^1^** $=\mathbf{(p}_{\mathbf{NCVD-NCVD}}\boldsymbol{)}$

The transitions from NCVD to CVD is given by:

**M^2^** $=\mathbf{(p}_{\mathbf{NCVD-CVD}}\boldsymbol{)}$

The probability of a 480-month-old adult free of CVD to the first transition into CVD after $k$ time steps (age groups) is the sum of the two non-zero entries of the first column of the following matrix:

**M^2^** $\boldsymbol{\cdot}$ **(M^1^) ^k-1^**

This calculation is performed for all the 241-time steps (age groups). Not all adults will transition into the state of CVD. Therefore, the sum of all 241 probabilities is less than one. This fraction represents the lifetime risk of CVD, i.e., the probability of ever experiencing CVD (Kemeny and Snell 1983; Mehta and Myrskylä 2017). To obtain the mean age at onset of CVD, we divide these probabilities by the lifetime risk so that they are summed to one and a proper distribution over the ages is obtained. They are used as weights for the ages to calculate the mean age at onset of CVD (Mehta and Myrskylä 2017).

**Calculation of the expectancies**

To obtain the expectancies with and without CVD, we first construct a matrix called the fundamental matrix, **F**:

$$\mathbf{F}=\left( \mathbf{I}-\mathbf{M} \right)^{-1}$$

= $\left( \begin{matrix} \mathbf{q}_{\mathbf{NCVD-NCVD}} & \boldsymbol{0} \\ \mathbf{q}_{\mathbf{NCVD-CVD}} & \mathbf{q}_{\mathbf{CVD-CVD}} \end{matrix} \right)$

where **I** is an identity matrix and **M** is a submatrix of **M^T^** containing only the transient states (NCVD and CVD):

$$\mathbf{M}=\left( \begin{matrix} \mathbf{p}_{\mathbf{NCVD-NCVD}} & \boldsymbol{0} \\ \mathbf{p}_{\mathbf{NCVD-CVD}} & \mathbf{p}_{\mathbf{CVD-CVD}} \end{matrix} \right)$$

Each entry ($\mathbf{q}_{\mathbf{i-j}}$) in the fundamental matrix $\mathbf{F}$ shows the probability of being in a particular age-health state combination before dying. When multiplied by three (3 x $\mathbf{q}_{\mathbf{i-j}}$**)** (we are using a quarterly-spaced model), it gives the expected duration of stay in that age-health state combination. Summing the entries of the first column of $\mathbf{q}_{\mathbf{NCVD-NCVD}}$ and then multiplying by three gives us the CVD-free expectancy for adults CVD-free aged 480 month. Likewise, adding the first columns of $\mathbf{q}_{\mathbf{NCVD-CVD}}$ and multiplying by three we get the CVD expectancy for adults CVD-free aged 480 month. Adding these two numbers gives the total life expectancy for adults CVD-free aged 480 month. We repeat the same calculations using the matrix block $\mathbf{q}_{\mathbf{CVD-CVD}}$ to obtain the CVD expectancy or total life expectancy for adults with CVD aged 480 month.

***Conditional and unconditional expectancies***

The expectancies obtained without applying weights (as discussed above) from the fundamental matrix are called conditional expectancies. For instance, CVD expectancy for adults CVD-free aged 480 month and for adults with CVD aged 480 month. To get the weighted expectancies, that is, CVD expectancy for an average adult aged 480 month, we apply weighted averages across the conditional expectancies. The weights are the starting state distributions in states with and without CVD aged 480-490 months. For each of the subpopulations, we use their own starting state distributions.


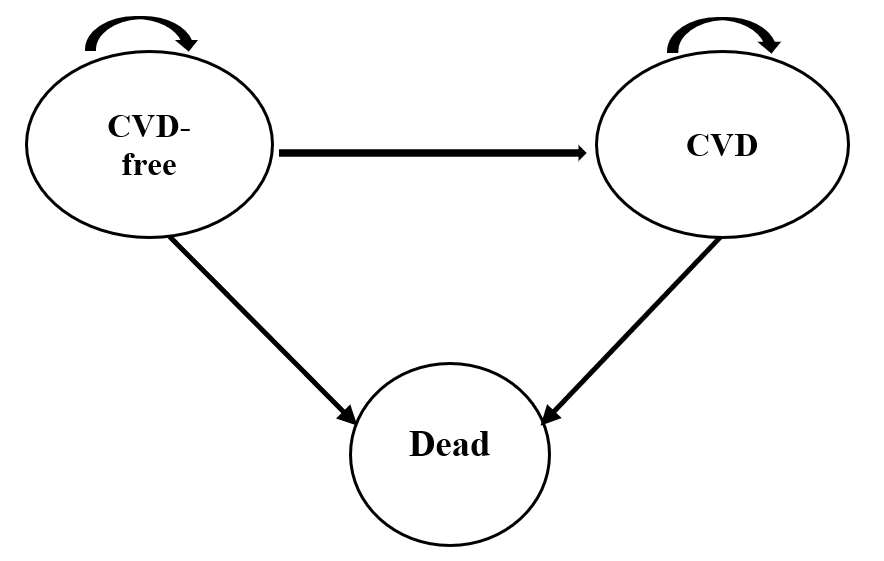


**Supplementary Figure 1**. The three states in the Markov state space

Note: CVD, cardiovascular disease

**Trends in the age-specific probabilities of transition from no cardiovascular disease to cardiovascular disease**

Supplementary Figure 2 shows the trends in the age-specific transition probabilities to the state of CVD for Finnish men and women. The age-specific transition probabilities have declined for both men and women across the study period, with the former experiencing a greater decline. In particular, the differences between 1996-2000 and 2016-2020 have widened at older ages, indicating that older men and women have experienced considerable reductions in the likelihood of transitioning to the state of CVD. However, at each period and for most of the age range, the transition probabilities have been greater among men than women. We observe a cross-over around the age of 91 years. That is, after the age of 91, the transition probabilities have been greater among women than men.

**Supplementary Figure 2.** Age-specific transition probabilities to the state of cardiovascular disease for men and women in Finland

**Trends in the age-specific probabilities of transition from cardiovascular disease to death**

Panels A and B in Supplementary Figure 3 show the trends in age-specific transition probabilities from CVD to death for Finnish women and men, respectively. At each period, men experience greater probabilities of transition from CVD to death than women. However, men have experienced slightly greater decline in these transition probabilities across the periods, narrowing the gender disparities.

**Supplementary Figure 3.** Age-specific transition probabilities from cardiovascular disease to death for women (Panel A) and men (Panel B) in Finland
